# Reoperation in Chiari-1 Malformations

**DOI:** 10.3390/jcm12082853

**Published:** 2023-04-13

**Authors:** Giuseppe Talamonti, Marco Picano, Maria Fragale, Eleonora Marcati, Giulia Meccariello, Davide Boeris, Marco Cenzato

**Affiliations:** 1 Department of Neurosurgery, ASST Niguarda, 20162 Milan, Italy; 2 La Sapienza, University of Rome, 00185 Rome, Italy

**Keywords:** Chiari malformation, CSF-leak, complications, foramen magnum decompression, reoperation, syringomyelia

## Abstract

(1) Background: The issue of unsuccessful surgery for Chiari-1 malformation (CM-1), as well as its potential causes and possible solutions, remains poorly documented and studied. (2) Methods: From a retrospective review of a personal series of 98 patients undergoing treatment for CM-1 during the past 10 years, we created two study groups. Group 1: 8 patients (8.1%) requiring additional surgeries owing to postoperative complications (7 cerebrospinal fluid leakage, 1 extradural hematoma); 7 patients (7.1%) undergoing reoperations for failed decompression during the follow-up. Group 2: During the same period, we also managed 19 patients who had previously been operated on elsewhere: 8 patients who required adequate CM-1 treatment following extradural section of the filum terminale; 11 patients requiring reoperations for failed decompression. Failed decompression was managed by adequate osteodural decompression, which was associated with tonsillectomy (6 cases), subarachnoid exploration (8 cases), graft substitution (6 cases), and occipito-cervical fixation/revision (1 case). (3) Results: There was no mortality or surgical morbidity in Group 1. However, one patient’s condition worsened due to untreatable syrinx. In Group 2, there were two cases of mortality, and surgical morbidity was represented by functional limitation and pain in the patient who needed revision of the occipitocervical fixation. Twenty patients improved (58.8%), 6 remained unchanged (32.3%), 1 worsened (2.9%) and 2 died (5.9%). (4) Conclusions: The rate of complications remains high in CM-1 treatment. Unfortunately, a certain rate of treatment failure is unavoidable, but it appears that a significant number of re-operations could have been avoided using proper indications and careful technique.

## 1. Introduction

Chiari malformation Type 1 (CM-1) consists of the downward displacement of the cerebellar tonsils into the spinal canal. Such ectopic tonsils do not necessarily constitute pathology. The wide diffusion of magnetic resonance imaging (MRI) is now showing a very high number of wholly asymptomatic and not progressive CM-1s [1,2,3,4,5,6,7]. These incidentally found CM-1s pose a problem with differential diagnosis and proper treatment indications [4,6]. There is general agreement that surgery should be reserved for symptomatic patients, but the correct assessment of preoperative symptoms may not be easy [2,6,7,8]. It is not fully understood why only some patients become symptomatic. It is supposed that symptoms and signs of CM-1 would depend on the ectopic tonsils hampering the free circulation of cerebrospinal fluid (CSF) between the cranial and spinal compartments [9]. The tonsils would isolate the perimedullary CSF space, which would become unable to dissipate the normal pressure increases that occur during exercise, Valsalva maneuvers, breathing, changes in head position and even systole [4,6,9]. This would create the conditions for the development of CM-1 symptoms and syrinx. The classic treatment aims to restore the CSF flow through the foramen magnum [9]. Foramen magnum decompression (FMD) usually consists of occipital craniectomy and C1 laminectomy, with or without dural opening, dural augmentation, Magendie exploration, or tonsillectomy. Patients with craniocervical joint malformations (CVJM) may need occipitocervical fixation, with or without transoral odontoidectomy [4,5]. Postoperative complications mainly consist of cerebrospinal fluid (CSF) leaks and pseudomeningocele (PSMC), and range from 0% to 23%, depending on the surgical technique [10,11,12,13]. The success rate of FMD is variable, ranging from 70% to 90% [9,11]. Postoperative complications do not always require reoperation, and not all patients with failed first surgery may improve with new surgical procedures. 

Anyway, a significant number of CM-1 patients require reoperation for three main reasons: (1) wrong initial indication or surgical technique; (2) postoperative complication; (3) ineffective surgery that can be improved by a more invasive procedure.

In this study, we retrospectively reviewed the charts of 34 consecutive patients who required additional surgical procedures following surgical treatment for CM-1 during the last 10 years.

## 2. Materials and Methods

Over the period 2010–2020, 98 patients with Chiari malformation (CM) underwent FMD at the Niguarda Hospital of Milan, Italy. There were 53 males and 45 females. The average age ranged from 2 to 71 years (mean 32 years). Ten patients (10.2%) were aged less than 15 years. All patients were preoperatively evaluated by phase contrast cine-MRI to study the CSF flow and enhanced MRI to assess possible arachnoiditis. A complete spinal cord MRI was also obtained. There were 84 patients (85.7%) with CM Type 1 (CM-1), while CM Type 1.5 (CM-1.5) was identified in 14 patients (14.2%). There was no Chiari 0 [14]. In 28 patients (28.5%) that were studied by high-resolution computer tomography (CT) scans, more or less complex CVJMs were reported. In 12 cases of suspected instability (12.2%), flexion-extension CT was also performed. Syringomyelia was found in a total of 39 patients (39.7%). The FMD consisted of a small occipital craniectomy (usually 2 × 3.5 cm) and a laminectomy at C1 in all cases. Only eight cases also required partial C2 removal. Sixteen patients underwent FMD with dural weakening, but without dural sac augmentation (FMD-WDA), whereas 82 subjects were also managed by dural sac augmentation (FMD + DA). All patients with CM-1.5 underwent FMD + DA. In all cases, the decompression extended to the spinal cord caudal to the ectopic tonsils. Patients who did not have syringomyelia or arachnoiditis on their enhanced MRI were treated with extra-arachnoid decompression. Every effort was made to keep the arachnoid membrane intact, even though accidental violations were not uncommon. Whenever a syrinx was present, the Magendie foramen and fourth ventricle were routinely explored. In the case of arachnoiditis, arachnoid dissection with arachnoidolysis was performed in an attempt to restore the subarachnoid space. At the first-time FMD, no patient required tonsillectomy, which we generally reserve for cases such as those with very low-lying tonsils or patients with syringomyelia, in whom the CSF flow was intraoperatively deemed inadequate after the fourth ventricle exploration. One patient (1.1%) had documented preoperative craniocervical instability and required fixation. There was no case of significant anterior compression that required odontoidectomy.

Eight of the 98 patients (8.1%) experienced postoperative complications, requiring reoperation. These patients have been considered as Group 1A.

There was neither mortality nor permanent surgically related morbidity and all patients were discharged on average within 10 days post-surgery (range 5 to 59 days). Thereafter, they were followed as outpatients for a mean period of 3.8 years (ranging from 1 to 10 years).

Four of 98 patients (4.1%) required further surgeries for symptom persistence or recurrence during the follow-up. Three adjunctive patients (who had been operated on before 2010) also required reoperation for ineffective decompression during the same period. Therefore, a total of seven patients were required to undergo further surgical procedures for the failed initial FMD. These seven patients represent the Group 1B and could be classified as CM-1 in five cases and CM-1.5 in two cases. 

Meanwhile, we managed 19 patients who had previously been managed at other hospitals.

In eight of these 19 patients, the previous treatment was just the extradural section of the filum terminale (EDS-FT). We categorized these eight patients into Group 2A.

The remaining 11 patients had undergone FMD, which failed for different reasons. These 11 patients have been considered as Group 2B.

Therefore, an overall number of 34 patients required reoperation due to failed initial treatment of their CM-1 syndromes. There were 31 adults and three children of 2, 12, and 14 years of age. Syringomyelia was reported in seven patients (five with CM-1 and two with CM-1.5).

All these patients were re-studied by cerebral and whole spine MRI. All cases were routinely re-assessed by phase contrast cine-MRI to assess the foramen magnum CSF dynamics. When cranio-cervical instability was suspected postoperatively, morpho-dynamic radiographs were obtained. 

**Figure 1:**
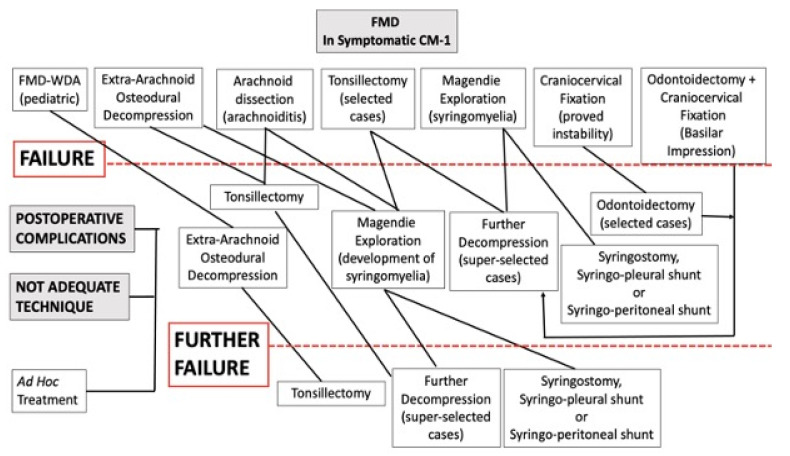
Schematic drawing of treatment for CM-1. The first line shows the initial surgical plan. The following lines describe the management of FMD failures. Postoperative complications and improper techniques were treated “ad hoc”. In the event of postoperative instability, craniocervical fixation was also performed. FMD = foramen magnum decompression; FMD-WDA = foramen magnum decompression without dural augmentation.

Postoperative complications and improper surgical treatment were managed by addressed treatment (for example, dural repair in the case of CSF leakage or adjunctive decompression in the case of inadequate bone removal). Patients who had been managed by FMD-WDA underwent FMD + DA. Patients with initial arachnoid preservation were managed by arachnoidolysis and tonsillectomy. First, the arachnoid adhesions around the tonsils were carefully dissected. Thereafter, a subpial tonsil resection was performed with care to preserve the posterior inferior cerebellar artery. A gentle, gradual tissue removal allowed for increased mobility and upward retraction of the pial surface of the tonsils, thus facilitating further arachnoid dissection. When free CSF flow was visible through Magendie’s foramen, a wide space was created around the bulbo-cervical junction, and when the reduced tonsils were rostral to the foramen magnum, the resection was deemed adequate. Syringomyelic cyst drainage was only used as a last resort. The algorithm for treatment is shown in Figure 1.

At the last follow-up, the clinical outcome of these patients was evaluated using the Chicago Chiari Outcome Scale [15]. Furthermore, the patient outcome was compared with the preoperative clinical state and was categorized as improved, unchanged, or worsened, based on the quality of life (QOL) [16].

The statistical analysis was conducted using the Fisher exact test or the Pearson chi-square test. 

The main features of these 34 patients are summarized in Table 1.

### 2.1. Group 1A (Postoperative Complications)

This group consisted of eight patients who had been treated with FMD + DA. There were seven adults and one child (of 2 years of age). Two days after surgery, a 32-year-old man experienced a worsening headache and a swollen occipitocervical wound. A CT-scan revealed an extradural clot that was immediately removed. At the reoperation, a bleeding vein was found close to the edge of the C1 laminectomy. The postoperative course was uneventful, and the patient was discharged seven days later without any new neurological deficit.

**Figure 2:**
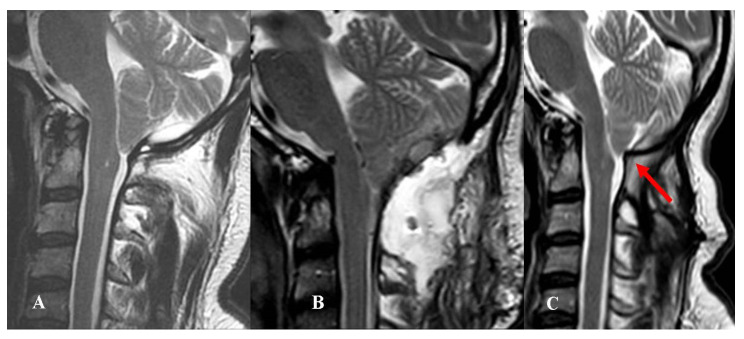
This 48-year-old man complained of an invalidating Valsalva-related headache. (**A**) Preoperative MRI, T2-weight, sagittal view showing a CM-1.5. Osteodural decompression was performed, and the arachnoid was accidentally violated. The dural sac was repaired using biological graft and fibrin glue. (**B**) Postoperative MRI, T2-weight, sagittal view showing a deep extradural pseudomeningocele that was not evident at skin level. Conservative treatment was attempted at first. The headache continued to worsen, especially in orthostatism, and 4 months later, he underwent reoperation for duraplasty using autologous fascia lata. (**C**) Follow-up MRI, T2-weight, sagittal view obtained 9 months after reoperation. There was no evidence of CSF collection; however, the orthostatic pain improved, but the headache did not yet resolve. Adhesions may have developed between the autologous graft and the cerebellum (red arrow). A further surgical procedure was offered, but was refused by the patient. The final CCOS score was 12.

Among the remaining seven patients, two experienced CSF wound leakage and five PSMC with dry skin wound. In four of five cases, the PSMC remained under the muscular layer and no subcutaneous fluid collection was evident (Figure 2).

The two patients with CSF leaks and the one with evident subcutaneous fluid collection underwent revision surgery, whereas the four patients with “internal” PSMC were initially conservatively managed. Since their preoperative headache continued or worsened, they were finally reoperated (7 days to 5 months after the first procedure).

Four of these seven patients had been treated with synthetic dural substitutes (polyurethane), while three had received biologic (bovine pericardial) substitutes. The new dural repair was accomplished using modern biologic dural substitutes (Lyoplant^®^, Aesculap AG- B-Braun, Tuttlingen, Germany) in the four cases initially managed using polyurethane graft. Conversely, autologous fascia lata was used in the three cases that had previously been managed using bovine pericardial grafts. In all cases, the suture lines were sealed using polyethylene glycol hydrogel sealant (DuraSeal^®^, Integra LifeScience Corporation, and Princeton, USA) and an adjunctive protective layer was created using gelfoam and fibrin glue. In five of the cases, an external lumbar drainage was postoperatively maintained for 5–7 days. One of these patients developed hydrocephalus, which was treated with a ventriculoperitoneal shunt.

### 2.2. Group 1B (Patients with Failed FMD in Our Series)

**Figure 3:**
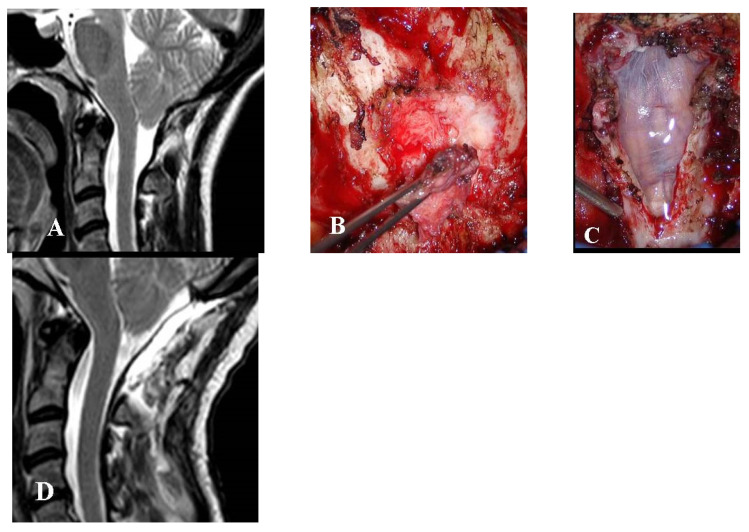
This 41-year-old woman had undergone bone decompression alone. Over the next 8 years, her headache recurred and progressively worsened. She also complained of dizziness and bouts of diplopia. (**A**) Preoperative MRI, T2-weight, sagittal view showing the bone decompression with scarce CSF film around the CM-1. (**B**) Intraoperative picture showing a thick and hard extradural fibrous scar that was maintaining the compression. (**C**) Intraoperative picture following dural opening with intact arachnoid. The dural sac was repaired using biologic graft. (**D**) Follow-up MRI, T2-weight, sagittal view obtained 6 months later showing the CM-1 was well bathed in CSF. Preoperative symptoms were almost completely resolved. The final CCOS score was 14.

This group consisted of six adults and one boy (14 years of age) who received limited or no benefit from FMD. All of these patients were re-admitted 6 months to 8 years after surgery. One of these patients had even undergone EDS-FT (at another center), six months after the failed FMD. All of these patients complained of the persistence or recurrence of their preoperative symptoms (headache in all cases, impaired left hand in one case). Four patients, three adults, and one boy (14-year-old), with FMD-WDA underwent reoperation for dural sac augmentation (Figure 3).

The remaining three patients had undergone FMD + DA; two experienced a progressive worsening of their preoperative symptoms despite adequate osteodural decompression. Both patients harbored CM-1.5. Radiological assessments did not reveal signs of instability, while phase contrast cine-MRI showed inadequate CSF-flow at the foramen magnum level. The reoperation of these two patients involved an extended arachnoid dissection and tonsillectomy. Six months after surgery, an adjunctive patient with FMD + DA presented with worsened syringomyelia and asymptomatic mild ventricular dilatation. Imaging studies did not reveal neural compression, craniocervical instability, or hampered CSF flow. This patient underwent surgical exploration with tonsillectomy. Since the syrinx continued to worsen, further procedures were required including ETV, ventriculoperitoneal shunting, further exploration with syringostomy, and finally syringoperitoneal derivation.

### 2.3. Group 2A (Patients Previously Undergoing Section of the Filum Terminale)

This group consisted of eight patients (seven adults and a 12-year-old girl) who had been managed by the EDS-FT at other hospitals. These patients were referred to us 6 months to 5 years after this treatment. Neither of these patients received any benefit from EDS-FT. Indeed, five patients with previous EDS-FT and subsequent FMD at other centers were assigned to Group 2B. Our treatment included FMD + DA in all patients of this group.

### 2.4. Group 2B (Patients with Ineffective FMD at Other Centers)

**Figure 4:**
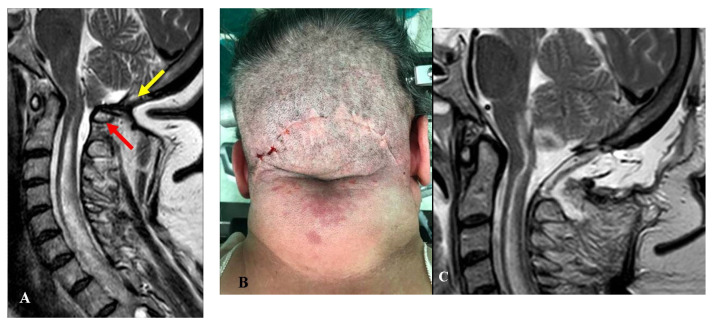
This 64-year-old woman had been treated with osteodural decompression, leaving C1 intact. One year later, she was referred to us for severe nocturnal breathing disturbances and mild quadriparesis. (**A**) Preoperative MRI, T2-weight, sagittal view, showing a small craniectomy with C1 still maintaining a significant obliteration of the arachnoid space (red arrow). A wide syringomyelia was evident. You will notice a marked skin retraction over the craniectomy (yellow arrow). (**B**) Intraoperative picture showing an “arc” incision and the skin retraction. The reoperation consisted of C1 laminectomy, tonsillectomy, and dural sac augmentation. (**C**) Postoperative MRI, T2-weight, sagittal view showing a relatively large subarachnoid space and initial syrinx shrinkage. There was mild local cerebellar edema owing to the tonsillectomy. An asymptomatic pseudomeningocele was also evident, which resolved spontaneously within a couple of weeks. Since the first postoperative period, breathing disturbances have significantly improved, whereas quadriparesis improvement has been slower. The final CCOS score was 10.

This group consisted of 11 adult patients. Six patients showed transitory improvement followed by symptom recurrence, whereas the remaining five received no benefit at all. This group included patients who had undergone different treatments. There were three patients with FMD-WDA who were reoperated for dural augmentation. Three other patients had been managed by FMD + DA, leaving C1 intact, which maintained a certain level of compression. The reoperation consisted of laminectomy and intradural exploration (Figure 4).

**Figure 5:**
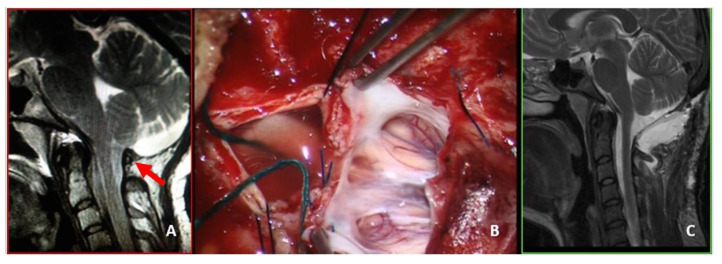
This 29-year-old woman had undergone osteodural decompression without laminectomy. One year later, she was admitted to our department with an untreatable persistent hiccup responsible for difficult alimentation, invalidating headache, dizziness, tinnitus, fever, rigor nucalis, and diffuse asthenia. An infection was suspected, but was ruled out. (**A**) Preoperative MRI, T2-weight, sagittal view showing a relatively wide craniectomy with C1 (red arrow) still compressing the arachnoid space, and a scarce presence of CSF around the CM-1. She underwent laminectomy and dural sac augmentation. (**B**) Intraoperative picture depicting a diffuse, tight adherence between the dural graft and the parenchyma. To replace the graft, a meticulous dissection was necessary. Finally, the dural sac was repaired with a synthetic graft. (**C**) Postoperative MRI, T2-weight, sagittal view showing a well-bathed CM-1. An asymptomatic pseudomeningocele was detected. This remained completely asymptomatic, even though it was still evident (but reduced) 6 months later. One year after the reoperation, the patient reported sporadic headache bouts, but had no other symptoms. The final CCOS score was 13.

Two other patients presented with worsening headache and rigor nucalis following a transitory improvement. These patients had undergone FMD + DA with biological meningeal grafts. MRI scans showed meningeal enhancement and reduced subarachnoid space with scarce CSF-flow in both cases. The reoperation consisted of graft substitution and arachnoid dissection (Figure 5).

Another patient received no benefit from FMD + DA. The repeated MRI revealed a partial dural ossification with limited craniectomy. Reoperation consisted of the enlargement of the osteodural decompression.

**Figure 6:**
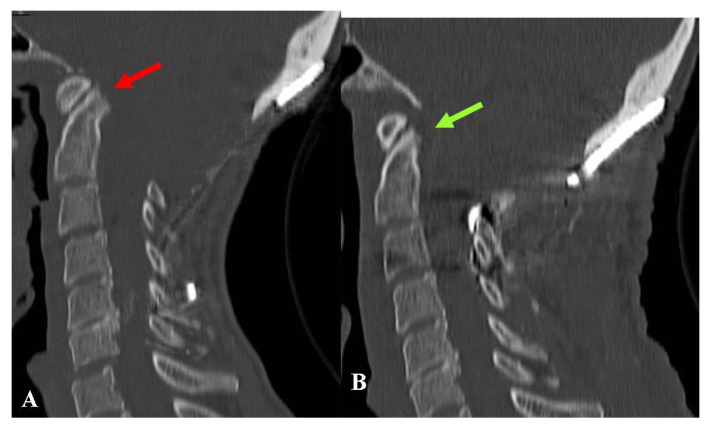
This 43-year-old woman had undergone osteodural decompression and craniocervical fixation. Six months later, she was referred to us due to progressively worsening swallowing disturbances, severe weight loss, asthenia, and marked continuous occipital pain. (**A**) Preoperative CT-scan, sagittal reconstruction showing a relatively wide bone decompression and a basilar impression with a quite acute clivo-axial angle (red arrow). Under neurophysiologic and radiologic monitoring, the patient underwent the removal of the fixation device and cranial traction. The basilar impression could be reduced, which made the planned procedure of odontoidectomy unnecessary. New craniocervical fixation devices were placed to maintain the new position. (**B**) Follow-up CT-scan, sagittal reconstruction, obtained one year later, showing resolution of the basilar impression (green arrow). The swallowing problems almost completely went away, and the headache got better, but the asthenia stayed. The final CCOS score was 10.

Another patient presented to us with severe dysphagia. She had been operated for FMD + DA plus occipito-cervical fixation. Repeated imaging studies documented ventral bulbar compression by basilar impression. The clivo-axial angle was below 135°. The treatment consisted of removal of the fixation device followed by cranial traction under general anesthesia with muscle relaxation under continuous radiologic and neurophysiologic monitoring. This enabled the basilar impression to be reduced. The reduction was maintained by placing a new craniocervical fixation (Figure 6).

Finally, there was a patient who had undergone several surgeries, including FMD + DA, transoral odontoidectomy, tonsillectomy, and craniocervical fixation. He was admitted to our department because of invalidating occipital and neck pain, breathing difficulties, and severe nocturnal apnea. He also suffered from quadriparesis. An MRI showed the enlargement of his known syringobulbia. In this case, we attempted to expand his osteodural decompression and explored the Magendie foramen to assess the patency of the fourth ventricle outlet.

## 3. Results

In addition to the eight patients from Group 1A and the seven from Group 1B, there were six patients who received no gross benefit from initial surgery and three subjects who developed worsening symptoms during follow-up but did not undergo further surgeries. Accordingly, a total of 24 patients (24.5%) did not improve after the first FMD. Although 13 of these patients improved following redo surgery, there were still 11 patients (11.2%) whose QOL did not improve (final CCOS < 13).

Surgical complications (Group 1A) were mainly surgeon-related, consisting of failed dural repair. On the other hand, several factors were responsible for FMD failure in Group 1B. However, there were no significant differences between the subtypes of Chiari malformation: two failures among 84 patients with Chiari malformation type 1 versus two failures among 14 patients with Chiari malformation type 1.5 (*p*-value > 0.05). In Groups 2A and 2B, which consisted of patients who had previously been operated on elsewhere, the weight of the subtypes was not evaluated.

The outcome of the 34 patients who required reoperation was evaluated, considering their initial status before any surgical treatment [15,16]. The QOL improved in twenty patients (58.8%), who scored 13–14 on the CCOS. Eleven patients (32.3%) had an unchanged QOL with a CCOS score of 10–12. One patient (2.9%) worsened (CCOS score = 4) and two died (5.8%) (Table 1 and Table 2).

Syringomyelia was the leading cause of worsening in all three patients, with negative results. In two of the remaining four patients with syrinx, the final QOL improved and remained unchanged in the other two. Accordingly, reoperation did not improve the QOL in five out of seven patients with syringomyelia, but these findings were not significant (*p* > 0.05).

The overall outcome was significantly better following first-time FMD than after redo surgery (*p*-value = 0.004).

Table 2 summarizes the outcome differences between the first and second operation.

Among the 20 patients with improved QOL following redo surgery, 17 (85%) were reoperated within 6 months, whereas 10 out of 14 patients without improved QOL underwent reoperation more than 6 months after the first surgery. The interval between the first and second operation was significant (*p*-value = 0.049).

### 3.1. Group 1A (Postoperative Complications)

The one patient with an extradural clot and the two patients with CSF leaks were successfully treated (final CCOS 14, 13, and 13). Upon reoperation, all five cases of PSMC were radiologically cured. Nevertheless, headaches persisted in three patients (final CCOS 12, 12, 10) (Figure 1) and improved in just two subjects (final CCOS 13 in both cases). These three patients without improvement underwent reoperation 2 to 5 months after FMD, whereas both patients with improvement were operated within one month of the first surgery.

Overall, 62.5% (5 out of 8) of patients achieved significant improvement, whereas three cases remained unchanged.

### 3.2. Group 1B (Patients with Failed FMD in Our Series)

All four patients with previous FMD-WDA improved following reoperation with dural augmentation (CCOS 14 in one case and 13 in three cases). As well as an extradural thick scar, fibrous extra-arachnoid bands were also found underneath the dural layer in all these patients. The subarachnoid space looked constricted and tended to expand as soon as the intradural bands were incised.

For the three patients who required reoperation with tonsillectomy, the following CCOS were obtained: 13, 10, and 4. The patient with a final CCOS score of 4 required numerous further surgical procedures, but continued to worsen and eventually became quadriplegic.

In conclusion, the QOL improved in five patients (71.4%), remained unchanged in one, and was severely worsened in another.

### 3.3. Group 2A (Patients Previously Undergoing Section of the Filum Terminale)

In six out of eight patients with previous EDS-FT, FMD + DA provided favorable clinical results (CCOS score 14 in two cases and 13 in four cases). Two patients obtained scarce clinical benefit (CCOS score respectively 11 and 10). Both of these patients had very long histories and were experiencing progressively worsening syringomyelia. They were referred to us more than 4 years after their EDS-FT. The FMD stopped the progression of the syrinx, but had a poor impact on headache and neurological status.

Overall, the QOL improved in six out of eight patients (75%), whereas it remained unchanged in two patients.

### 3.4. Group 2B (Patients with Ineffective FMD at Other Centers)

Among the three patients who had undergone FMD-WDA, two improved following dural augmentation (CCOS score respectively 14 and 13), while one obtained just transitory benefit and required further surgery consisting of bilateral tonsillectomy, which provided just partial improvement (CCOS score = 12).

One of the three patients with C1 intact also had very tight adhesions between the cerebellar cortex and the overlying biologic dural graft, which had to be meticulously detached and replaced using an inert dural substitute of polyurethane (Figure 5). These three patients experienced partial improvement, but their QOL remained unchanged (final CCOS = 12 in one case and 10 in two cases).

The outcomes of those two patients with foreign body reactions to the dural substitute were quite different. One significantly improved by dural graft replacement and achieved a CCOS score of 13. The other patient had a long clinical history of arachnoiditis and had only mild and transitory improvement. Despite numerous further surgical procedures, this patient progressively became quadriplegic and eventually died because of syrinx progression and final bulbar atrophy.

Regarding the patient with craniocervical fixation and basilar impression (Figure 6), postoperative assessments demonstrated an adequate reduction in the bone malformation. Although her swallowing disturbances improved, one year later she still complained of severe headache and asthenia. The final CCOS score was 10 and her QOL was unchanged.

Following reoperation, the patient with limited craniectomy achieved a good QOL (final CCOS score = 13).

The patient who had undergone multiple procedures for FMD-DA, odontoidectomy, craniocervical fixation, and tonsillectomy had an uneventful course following reoperation. Postoperative MRI showed a decrease in syringobulbia. However, one week later, this patient complained of severe vegetative disturbances and ultimately died despite no new lesion being shown by repeated MRI. This patient has already been reported [4].

In conclusion, in these 11 patients, the QOL improved in four and remained unchanged in five. Two patients died.

## 4. Discussion

Reoperation due to failed treatment is a major problem in the management of CM-1 syndrome. All series show rates of treatment failures that are not negligible [7,11,17,18,19,20,21,22,23,24,25,26]. However, the skillfulness of the surgeon may play a significant role: in the series of Walker-Palmer et al. [26], the complication rate ranged from 11 to 20% for extradural, and 10.5 to 40% for intradural surgeries depending on the experience of the surgeons. Basaran and Colleagues [27] reported a higher rate of complications in CM-1.5 than in CM-1. This finding was not confirmed by Chae and Greenfield [24], who reported no significant differences in the rate of reoperations. Patients with CM-1.5 would have more severe syndromes and shorter clinical histories [27] and may require different treatment modalities [14]. However, there are authors [24,27] who still consider FMD with or without DA to be the preferred procedure for both CM-1 and CM-1.5. In our series, syringomyelia was significantly more frequent in CM-1.5 patients, but CM-1 and CM-1.5 had comparable rates of treatment failures. We usually extend the decompression to expose the space caudal to the tonsils, even if this means a partial C2 laminectomy, which is one of the recommended treatments for CM-1.5 [28] and for syringomyelia [29].

Recently, Oldfield and colleagues [9,30,31] reviewed their extensive 30-year experience reporting the results of the various surgical techniques that can be chosen. They reported some outcome differences between children and adults, and compared bone decompression alone with dural augmentation. They found a lower complication rate, but a higher probability of reoperation, in patients undergoing bone decompression alone. Conversely, patients with osteodural decompression required fewer surgeries overall, despite higher rates of postoperative complications. Similar findings were also reported by others [26,29]. A generally accepted strategy is to perform bone decompression alone with dural weakening in cases with less severe malformations [32] and in the pediatric population [9,30,33,34,35]. The presence of syringomyelia is often considered an indication to perform dural augmentation, and subarachnoid exploration to identify arachnoid webs or veils impairing the flow from the 4th ventricle [3,6,29,36]. Other authors [26] reported no significantly different results comparing extra- and intradural procedures in the management of pediatric syringomyelia.

Klekamp [25] found a close correlation between the severity of neurological symptoms and the grade of arachnoid pathology, and recommended decompressions with arachnoid dissection to obtain a favorable long-term prognosis. This author, however, reported that first-time decompression with arachnoid dissection resulted in surgical morbidity of 2.0%, a 0.9% mortality rate. We have recognized the importance of arachnoiditis since 1993 [37]; however, in patients without evidence of arachnoid pathology and/or syringomyelia, we prefer extra-arachnoid decompression as initial treatment. This should reduce the surgical risks. During first-time FMD, we do not even routinely perform tonsillectomy. Some authors [38,39] still perceive tonsillectomy to be the most effective treatment modality, whereas others [35] dispute the efficacy, safety, and necessity of tonsillar manipulation. Although techniques exist for mini-invasive tonsillectomy, in our and other minds [9,31,40,41], when it is possible, extra-arachnoid decompression is preferred, since adhesions and scarring are triggered each time the arachnoid is entered. In cases where a tonsillectomy is necessary, gentle coagulation of the tonsils should result in fewer adhesions and scarring. However, we prefer a subpial tonsillectomy, which may provide a wider space for the CSF. In our experience, the preservation of the pial surface usually warrants scarce arachnoid scars.

Dural repair is generally accomplished using commercial dural substitutes or autologous muscular fascia. A discussion of the best method of dural reconstruction is beyond the scope of this paper, and a gold standard does not exist for this item either [35,36,37,38,39]. Inert artificial grafts, such as those in polyurethane or Gore-Tex^®^ (Gore, DE, USA), would warrant no cerebellar-arachnoid adhesions, thus favoring free CSF circulation and improving patient outcomes. On the other hand, these grafts also provide less adhesion with the dural boundaries, thus promoting less waterproof healing and a relatively higher risk of CSF leakage. Conversely, biologic grafts (both commercial and autologous ones) offer a lower risk of CSF fibula but a higher risk of internal adhesions hindering the CSF circulation. Even though the international consensus document [40,41] does not favor the use of inert artificial grafts, considering the above general aspects, we choose the dural graft case by case.

An international consensus conference was recently held in Milan, Italy to reach a consensus about the indications and the management of CM-1 [37,38,39]. However, several authors maintain their preferences and a gold standard does not yet exist [35,38,39,40,41].

Further surgeries owing to FMD failure are reported in 6.6–21.6% of patients [20,26,42,43]. In our series, 7.1% of patients required reoperation because of failed first-time FMD, and 11.2% did not achieve any improvement in their QOL (CCOS < 13). We strive to improve our results following most of the recommendations of the international consensus document [40,41] that we contributed to carrying out. FMD-WDA is now offered only in selected cases of children without syringomyelia. The arachnoid membrane is preserved in patients without arachnoiditis or syringomyelia. Tonsillectomy is performed only in patients with very low-lying tonsils or with seriously hampered CSF flow from the fourth ventricle. Asymptomatic and paucisymptomatic PSMC are initially conservatively treated. Symptomatic basilar invagination is managed by anterior decompression when posterior reduction has already failed, and so on. Nevertheless, in some cases, our policy may differ from the consensus indications. For example, we do not hesitate to extend bone decompression up to C2 in the case of low-lying tonsils.

There are a few papers focusing on the results of reoperations in CM-1 patients [24], and we can provide limited information regarding the overall risk of complications, the rate of reoperation, and the outcome of reoperated patients. Of the 34 reoperated patients in our series, none achieved the maximum CCOS score of 16 and 41.2% obtained unsatisfactory results (CCOS score < 13). Furthermore, reoperation resulted in significantly worse results than first-time FMD. We cannot state that surgery for CM-1 should be considered a “one-shot-therapy”, but we think that any effort must be made to have the first surgery as effective as possible. This statement may seem obvious, but we mean that all the procedures must be carefully evaluated and indicated, including bone decompression alone, preservation of C1, dural augmentation, extra-arachnoid decompression, and so on. Since long clinical histories may have a negative prognostic effect, it is important to avoid procedures of unproven efficacy (such as EDS-FT) since they may delay the proper treatment [40,41].

Syringomyelia played an important role in our series: it was responsible for one-fifth of patients worsening after the first treatment, and less than one-third of patients improved after reoperation. Furthermore, it was responsible for the three cases of poor results.

The sample size was too small for sophisticated statistical analyses. Regardless, these findings should be considered. Although the statistical significance of the presence of syringomyelia was not reached and was weak regarding delayed reoperations, poor results were found in syringomyelic patients who wasted their time with EDS-FT.

### 4.1. Group 1A (Postoperative Complications)

Our overall rate (7.6%) of patients requiring re-operation because of postoperative complications is not negligible but favorably compares with the 6%-7% rates from the literature [7,11,20,42,43]. Anyway, it should be noted that almost 80% of our patients underwent dural augmentation and most of our complications consisted of CSF leakage or PSMC.

Although the literature reports comparable rates (13%) of CSF-leak in all types of posterior fossa surgery [44,45], in our experience [46], the risk of CSF fistula appears higher in CM-1 treatment. We wonder if a role is played by the disturbed CSF dynamics of CM-1 syndrome [6,9].

Postoperative PSMCs with no external evidence, are not uncommon. If they are asymptomatic or paucisymptomatic, they can be treated conservatively [40,41]. A simple observation, head elevation, and sometimes temporary external lumbar drainage, can be quite effective. However, if the PSMC is large, this tends to increase or is associated with an important headache, then a timely surgical indication must be considered [40,41]. In the present series, headaches were frequent in PSMC, as also reported by others [47]. All the three patients who were lately re-operated had unchanged QOL. It is not possible to draw reliable inferences, but both patients with improved QOL were early operated.

### 4.2. Group 1B (Patients with Failed FMD in Our Series)

This group mainly consisted of adult patients requiring reoperation following FMD-WDA. All of these patients improved after dural sac augmentation. As mentioned above, we have stopped offering FMD-WDA to adult patients.

Arachnoiditis and foreign body reactions may be difficult to manage. Graft replacement and extended arachnoid dissection may provide only temporary benefit because arachnoid adhesions tend to recur. We usually perform tonsillectomy as well in these cases. This improves decompression and increases the space for CSF-flow. In general, we commonly perform tonsillectomy at the second-time FMD.

When clear causes of failed FMD are identified, the treatment may be relatively simple and satisfactory. However, this group included a case of FMD failure without any apparent cause. Despite no evidence of persistent compression, hampered CSF-flow, or instability, this patient progressively worsened owing to untreatable syrinx progression. We are still perplexed about this patient. In addition to compression, CSF flow alteration, and instability, there may be some other still unknown mechanisms that play a role in CM-1 syndrome.

### 4.3. Group 2A (Patients Previously Undergoing Section of the Filum Terminale)

The section of the filum terminale as treatment for CM-1 syndrome originates from the observation that tethered cord may be associated with CM-1 and that CM-1 may be an acquired condition in children with spinal dysraphism [48,49]. Indeed, such an association is not as frequent as originally reported [50]. Treatment should always address the symptomatic lesion: patients with tethered cord syndrome should undergo detethering, whereas patients with CM-1 syndrome should obtain cranio-cervical decompression [51]. Anatomical studies [52] have provided arguments against the role played by pulling-down in CM-1 development: both the cerebellum and the brainstem are frequently upwardly displaced through tentorial hypoplasia; cervical and thoracic roots always present normal origin and direction. Moreover, there are experimental studies denying any pathogenetic effect on CM-1 development by tethering the cord [53]. The extradural section of the filum terminale would mean the interruption of the so-called filum terminale externum, which simply connects the dural sac to the coccyx, and has no proven relationships with the filum terminale internum, which ends fusing with the dural sac [54]. From a practical standpoint, the theoretical traction by the filum terminale externum would be exerted on the dural sac, with no effect on the spinal cord. Thus, on the CM-1 development. Finally, the literature includes few, and poorly documented, references that support the efficacy of filum terminale sectioning. A recent systematic review found only two published series of EDS-FT, both from the same group, and concluded that this procedure has no scientific support in CM-1 patients without evidence of tethered cord [55].

The unique advantage of EDS-FT is that it is a real low-risk procedure. On the other hand, the EDS-FT may delay the proper treatment of CM-1, with possible negative effects. While the real efficacy of EDS-FT remains unproven [40,41], it is a matter of fact that 14 out of 34 patients (41.1%) with CM-1 requiring reoperation had been previously managed by filum terminale sectioning.

### 4.4. Group 2B (Patients with Ineffective FMD at Other Centers)

Some patients experienced temporary postoperative improvement. These patients were mainly adults with FMD-WDA or with arachnoiditis. On the other hand, there were patients without any postoperative improvement after FMD. In all these cases, the decompression was too small. It might be useful to categorize FMD failures.

It is widely accepted that a craniectomy of 2 × 3.5 cm is sufficient to treat most cases. If C1 does not participate in the compression, then it is also reasonable that it is preserved. However, our series included patients with small craniectomy and patients with untouched C1 despite its compressive effect. In general, careful analysis of the preoperative imaging is essential to plan an adequate surgical strategy that must be closely tailored to each patient. Another consideration concerns possible ventral compression. 

This series included a patient with misrecognized basilar impression, which was the cause of the failure. Odontoidectomy has been recommended in the case of CM-1 with ventral compression [5,56]. It has been recommended to try reducing the basilar impression before removing the dens [57]. We routinely perform odontoidectomy in the case of irreducible basilar impressions [40,41], but cases of CM-1 requiring odontoid removal were never encountered during the last 10 years. Odontoidectomy is often associated with cranio-cervical fixation. Some authors have stressed the indications for occipito-cervical arthrodesis, both as the first treatment for CM-1 and in the case of redo surgeries [32,58]. However, the AO-spine has considered craniocervical fixation as a morbidity [59,60], and its possible advantages must be carefully evaluated considering its undoubted disadvantages. In our own series, we only had to fixate one patient with preoperative proven instability, whereas no patient developed postoperative instability and required fixation on reoperation. This group included two patients who had been fixed elsewhere. Both patients complained of severe neck rigidity and pain, and asked for the fixation removed. In one case, the removal was absolutely impossible because of extended and consolidated arthrodesis [4]. The other case was that of the patient whose fixation device was temporarily taken off to reduce the basilar impression (Figure 5).

## 5. Conclusions

The need for repeated surgical procedures remains a major problem in CM-1 treatment. Several well-known factors can contribute to the failure of the initial treatment, the management of which is relatively well codified and accepted [37,38,39]. The small sample size of this series prevents definitive conclusions. The majority of patients had to be reoperated due to surgical complications or inadequate decompression, as well as inappropriate treatment choices. These factors are surgeon-related and need to be improved [26]. Other patients had to be reoperated because of arachnoiditis, syringomyelia, and associated CVJMs. These are disease-related factors that may be difficult to treat and require a correct diagnosis and adequate surgical planning and management. Therefore, in all cases, the importance of the surgeon’s skill and experience cannot be overemphasized, as they play a crucial role both in the prevention and management of CM-1 treatment failures.

Furthermore, it is advisable that patients who require redo surgery are managed without unmotivated delays, as long intervals between the first and second operation may affect the outcome.

Patients who required reoperation had overall worse outcomes than patients with effective initial treatment.

Therefore, every effort should be made to ensure that the first treatment is definitive. With the careful examination of the patients and their neuroimaging studies, it is possible to develop tailored strategies to increase the chances of surgical success.

Unfortunately, Chiari surgery is often considered routine and not taken as seriously and handled as delicately as other neurosurgical interventions. We have the impression that the CM-1 treatment is often considered relatively easy and, therefore, is assigned to less experienced surgeons. It appears that even the indications and techniques are sometimes chosen superficially, representing the best way to cause disasters.

We hope that this wrong perspective and perception will change and that we will train our young people to understand the importance of, and both the manual and intellectual delicacy needed in, Chiari surgery.

## Figures and Tables

**Table 1:** Main features of 34 patients undergoing reoperation for Chiari Malformation.

	Previous Treatment	Clinical Manifestations	Cause of Reoperation	Treatment	Outcome *			
					Improvement	No Change	Worsening	Death
**Gr. 1A** **8 pts**	**8** FMD + DA	**1** neck pain, wound swelling	**1** extradural clot	excision	**1** (14)			
		**2** CSF-fistula	**2** failed dural repair	dural repair	**2** (13-13)			
		**5** PSMC	**5** failed dural repair	dural repair/graft substitution, later VPS	**2** (13-13)	**3** (12-12-10)		
**Gr. 1B** **7 pts**	**4** FMD-WDA	**4** symptom recurrence	**4** failed extradural FMD	dural sac augmentation	**4** (14-13-13-13)			
	**3** FMD + DA	**2** symptom recurrence	**2** inadequate CSF flow (cine-MRI) arachnoiditis	arachnoid dissection tonsillectomy	**1** (13)	**1** (10)		
		**1** symptom recurrence, syrinx progression	**1** unknown cause (arachnoiditis?)	arachnoid dissection tonsillectomy later ETV, VPS, SPS			**1** (4)	
**Gr. 2A** **8 pts**	**8** EDS-FT	**6** symptom recurrence **2** symptom recurrence, syrinx progression	**8** improper treatment	FMD + DA	**6** (14-14-13-13-13-13)	**2** (11-10)		
**Gr. 2B** **11 pts**	**3** FMD-WDA	**3** symptom recurrence	**3** failed extradural FMD	dural sac augmentation	**2** (14-13)	**1** (12)		
	**3** FMD + DA with C1 intact	**2** symptom recurrence **1** symptom recurrence, syrinx progression	**3** compression by C1	C1 laminectomy, intradural exploration, tonsillectomy (in 1 case)		**3** (12-10-10)		
	**2** FMD + DA	**1** symptom recurrence **1** symptom recurrence, syrinx progression	**2** foreign body reaction/arachnoiditis	dural graft substitution, arachnoid dissection, tonsillectomy (in 1 case)	**1** (13)			**1** (4)
	**1** FMD + DA, cranio-cervical fixation	**1** symptom recurrence	**1** mis-recognized basilar impression	impression reduction by traction, new fixation		**1** (10)		
	**1** FMD-DA	**1** symptom recurrence	**1** limited craniectomy	osteodural decompression	**1** (13)			
	**1** FMD + DA, fixation, odontoidectomy	**1** symptom recurrence, syrinx progression	**1** limited craniectomy	osteodural decompression, tonsillectomy				**1** (4)
total	34		34		20 58.8%	11 32.3%	1 2.9%	2 5.9%

FMD + DA = foramen magnum decompression + dural augmentation; FMD-WDA = foramen magnum decompression without dural augmentation; CSF = cerebrospinal fluid; PSMC = pseudomeningocele; OBDA = occipital bone decompression alone; EDS-FT = extradural section filum terminale; MRI = magnetic resonance imaging; ETC = endoscopic third ventriculostomy; VPS = ventriculoperitoneal shunting; SPS = syringoperitoneal shunting; C1 = atlas. * The number of patients is reported in bold; the numbers in parentheses represent the CCOS score for each patient [15,16].

**Table 2:** Comparison between the outcomes of first-time and second-time surgeries. FMD = foramen magnum decompression; QOL = quality of life; CCOS = Chicago Chiari Outcome Scale [15,16].

	Improved QOL (CCOS 13–16)	Unchanged QOL (CCOS 10–12)	Worsened/Dead QOL (CCOS 4–9)	
First-time FMD 98 pts	87 (88.7%)	10 (10.2%)	1 (1.1%)	*p =* 0.004 (significant)
Redo Surgery 34 pts	20 (58.8%)	11 (32.4%)	3 (8.8%)	

## Data Availability

No new data were created or analyzed in this study. Data sharing is not applicable to this article.
